# MUCOCUTANEOUS CHANGES IN TUBEROUS SCLEROSIS COMPLEX: A CLINICAL PROFILE OF 27 INDIAN PATIENTS

**DOI:** 10.4103/0019-5154.55636

**Published:** 2009

**Authors:** Sudip Kumar Ghosh, Debabrata Bandyopadhyay, Gobinda Chatterjee, Arghyaprasun Ghosh, Sharmila Sarkar, Somenath Sarkar

**Affiliations:** *From the Departments of Dermatology, Venereology and Leprosy, R.G.Kar Medical College, Kolkata, India.*; 1 *From the Department of Psychiatry, Medical College Calcutta, India.*

**Keywords:** *Tuberous sclerosis*, *mucocutaneous*, *adenoma sebaceum*, *shagreen patch*

## Abstract

**Background:**

Tuberous sclerosis complex (TSC) is an autosomal dominant neurocutaneous disease resulting in a wide array of clinical manifestations, primarily affecting the skin and central nervous system. Mucocutaneous features play a very important role in the recognition of this syndrome.

**Aims:**

To review the prevalence and patterns of cutaneous manifestations in tuberous sclerosis, in a group of patients from eastern India.

**Methods:**

Observational clinical study on twenty-seven consecutive patients of tuberous sclerosis collected during a period of four years.

**Results:**

Most were between 10–20 years of age; the male to female ratio was 2:1. Family history was found in two-thirds. The classical triad of tuberous sclerosis was present in only nine (33.3%) patients. Adenoma sebaceum was the most common cutaneous feature (100%), followed by hypomelanotic macules (92.6%), connective tissue nevi (66.6%), and Koenen's tumors (33.3%). Oral mucosal fibromas were seen in six (22.22%) patients. Fibromatous plaque over forehead and scalp was seen in three patients. Limitation of the study was small size of study sample.

**Conclusion:**

Prominent mucocutaneous changes are extremely common manifestation of TSC, which may provide crucial diagnostic clues for primary care physicians.

## Introduction

Tuberous sclerosis complex (TSC) is an autosomal dominant neurocutaneous disease resulting from mutation of one of the two different genes (TSC-1 and TSC-2).[1] The disease primarily affects the skin and the central nervous system as well as other organ systems. There is also a predisposition to development of tumors. The diagnosis of the syndrome is essentially clinical, based on the presence of a constellation of various cutaneous and systemic changes. Comprehensive criteria exist for the diagnosis of the disorder.[2]

Although various combinations of clinical manifestations may occur, mucocutaneous lesions, by virtue of their easy visibility, are quite often the most important presenting feature of the disease. The aim of our study has been to review the prevalence and patterns of cutaneous manifestations among patients of tuberous sclerosis, in a population from eastern India. Absence of any formal study on this topic from this region prompted us to undertake the present work.

## Materials and Methods

It was an observational clinical study, carried out at a tertiary healthcare setup in Kolkata, West Bengal, India. The study was carried out over four years and 27 consecutive patients fulfilling the diagnostic criteria of tuberous sclerosis formed the study population. The patients attended the dermatology clinic during that period either directly or were referred from other departments of this hospital. A detailed history was taken and every patient was subjected to thorough clinical examination. Imaging and relevant laboratory investigations were done whenever needed.

## Results

Twenty-seven patients of tuberous sclerosis have been evaluated. All patients were lifetime residents of the eastern Indian state of West Bengal. Patients in their second decade formed the most common age group. Eighteen patients were men and nine were women with a male to female ratio 2:1. Positive family history was present in nine (33.3%) patients. Among the patients with positive family history, six had sibling afflictions and three had history of parental afflictions.

**Figure 1:**
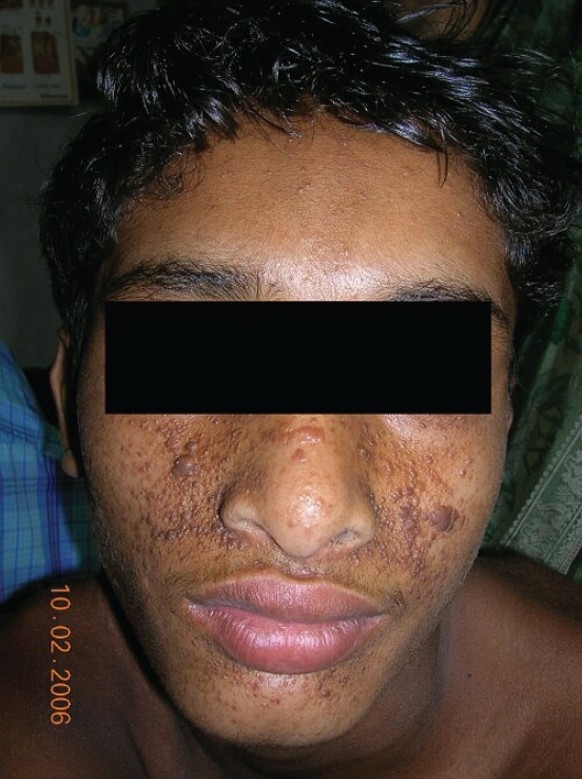
Multiple adenoma sebaceum on face

**Figure 2:**
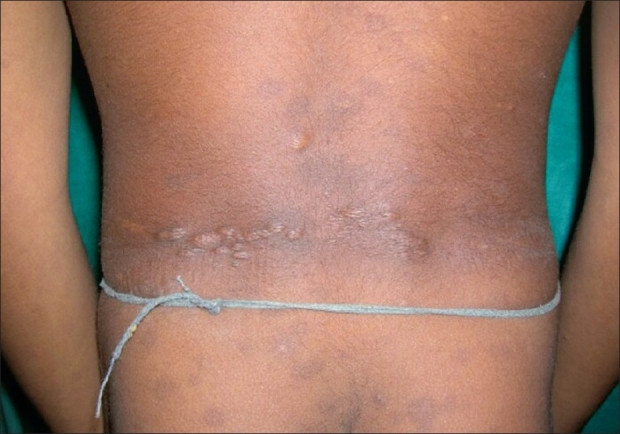
Shagreen patches on the back in a child

**Figure 3:**
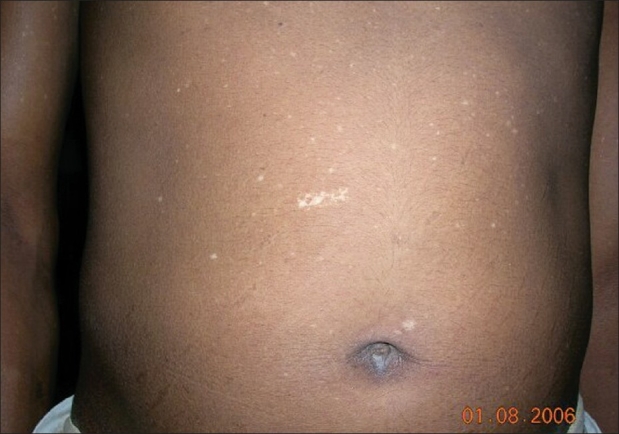
Multiple small hypopigmented ‘confetti’ macules on abdomen and upper limbs in a young boy

**Figure 4:**
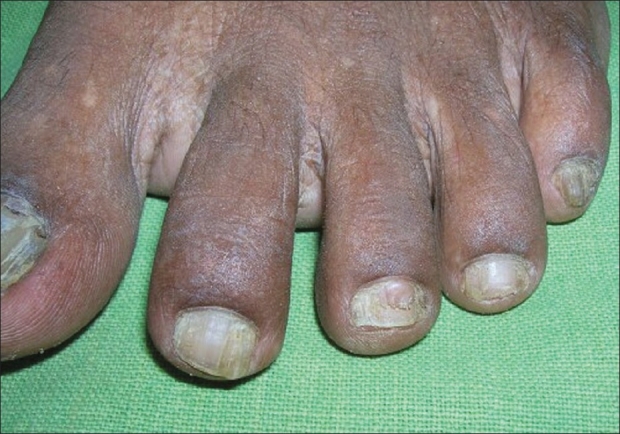
Subungual fibromas (Koenen's tumors) of toes

History of epilepsy was present in 18 (66.7%) patients and 13 (48.1%) had mental retardation. Adenoma sebaceum [Figure 1] was present in all (100%) patients. The classical triad of tuberous sclerosis (adenoma sebaceum, mental retardation, and epilepsy) was present in only nine (33.3%) patients. Regarding other cutaneous lesions [Table 1], connective tissue nevi was present in 18 (66.7%) patients, lower back (shagreen patch) being the commonest 14 (51.8%) site [Figure 2]. Hypomelanotic macules were found in 25 (92.6%) patients including confetti macules [Figure 3] in nine (33.3%) patients. Koenen's tumor was noted in nine (33.33%) patients [Figure 4]. Of these patients, one-third had only fingernail affliction, one-third had toe nail affliction, and the remaining had both toe and finger nail affliction.Fibromatous plaque over forehead and scalp was seen in three patients. Oral mucosal fibromas were seen in six (22.2%) patients.

**Table 1:** Mucocutaneous features of tuberous sclerosis (n = 27)

Mucocutaneous features	Number	Percentage
Adenoma sebaceum	27	100
Hypomelanotic macule	25	92.6
Connective tissue nevi	18	66.7
Koenen's tumor	9	33.3
Oral mucosal fibroma	6	22.2
Scalp and forehead plaque	3	11.1

## Discussion

Tuberous sclerosis complex is a neurocutaneous syndrome involving multiple organ systems and shows extremely variable clinical manifestations.[3] The two genes, TSC-1 and TSC-2, involved in disease manifestations, are located on chromosomes 9 and 16, respectively.[4] Since these genes act as tumor suppressors, their mutation leads to hamartomatous lesions in various organs including the skin. Although, focal or generalized seizures with or without developmental delay are often the first sign of the disease, careful examination can detect hypopigmented lesions even earlier, providing an early diagnostic clue.[5]

Tuberous sclerosis can present at any age.[6] Our patients most commonly presented in their second decades of life.The gender ratio in this study was 2:1 in favor of males, although literature shows the sex ratio to be almost equal with symptoms being more predominant in females.[5] On the other hand, some studies have suggested that men are more likely to suffer from neurological morbidity.[6] Positive family history was present in one-third of our patients and this result corroborates with prevailing data.[5] Epilepsy is a very important feature of tuberous sclerosis and some authorities recommend that all children with epilepsy be assessed for tuberous sclerosis.[7] Existing data states the incidence of epilepsy to be in the range of 70% to nearly 90%.[4,7,8] Epilepsy was present in 66.7% patients in the present study. Mental retardation can occur in 40-70% of patients of TSC.[4,8] Nearly half of our patients had this feature.

Tuberous sclerosis complex has a wide range of mucocutaneous manifestations that includes adenoma sebaceum (angiofibroma), connective tissue nevi, hypomelanotic macules, periungual fibromatous lesions, fibromatous plaque on forehead, and oral mucosal fibromas. Adenoma sebaceum is the best-known cutaneous manifestation of tuberous sclerosis. It is clinically characterized by multiple, discrete, translucent, reddish waxy papules that are distributed symmetrically around the nose and over the cheeks and forehead. Ninety to ninety six percent of the patients may develop adenoma sebaceum by the age of four years.[5,8] This feature was found in all (100%) the patients in the present study. Another Indian study has also described similar affliction (100%) with adenoma sebaceum.[9] The classical triad of tuberous sclerosis consisting of adenoma sebaceum, mental retardation, and epilepsy is said to be present in about one-third of patients.[5] The present study revealed a similar prevalence (33%). Hypomelanotic macules are commonly found in TSC and may take the forms of multiple small confetti-like lesions or elongated off-white lesions, the so-called ‘ash-leaf’ macules. These are nonspecific findings and are not pathognomonic of tuberous sclerosis.[6] They were present in more than 90% of patients including one-third of the patients with confetti macules. A wide range (60-90%) of frequency of affliction with these lesions is cited in the literature.[4,5,10] Connective tissue nevi are leathery plaques, found on various parts of body. The so-called shagreen patches are connective tissue nevi on lower back. This was observed in 66.7% patients, although they are stated to occur with much less frequency in Western literature (40%).[8] Another Indian study has also described a higher prevalence (80%) of shagreen patches.[9] Scalp and forehead plaques were found in 11% of our patients in comparison to a much higher percentage (47%) observed in a previous Indian series.[11] According to a study, forehead plaques may be considered to be a novel cutaneous marker of CNS involvement at an early stage of tuberous sclerosis.[11] Koenen's tumors, which are small digitate protruding asymptomatic periungual fibromas, were found in one-third of our patients. Oral mucosal fibromas were noted in 22.2% patients in this study, but one recent study reported a relatively higher percentage (69%) of affliction.[12]

Thus, the easily recognizable and distinct mucocutaneous features are very frequent findings of this syndrome and awareness and recognition of them may help physicians to diagnose this important neurocutaneous syndrome at an early state. However, a multidisciplinary approach must be undertaken from both diagnostic and therapeutic point of view. A limitation of the present study was that the study population was small and a larger sample size might highlight in a better way the prevalence and pattern of mucocutaneous manifestations of TSC.
